# Serum glycomic profile as a predictive biomarker of recurrence in patients with differentiated thyroid cancer

**DOI:** 10.1002/cam4.5465

**Published:** 2022-11-27

**Authors:** Matthew R. Kudelka, Yi Lasanajak, David F. Smith, Xuezheng Song, Mohammad S. Hossain, Taofeek K. Owonikoko

**Affiliations:** ^1^ Department of Medicine Memorial Sloan Kettering Cancer Center New York City New York USA; ^2^ Department of Biochemistry Emory University School of Medicine Atlanta Georgia USA; ^3^ Department of Hematology and Medical Oncology Emory University Winship Cancer Institute Atlanta Georgia USA; ^4^ Present address: Division of Hematology and Oncology University of Pittsburgh Pittsburgh PA USA

**Keywords:** biomarker, Glycome, glycan, profiling, recurrence, thyroid cancer

## Abstract

**Purpose:**

Thyroid cancer recurrence following curative thyroidectomy is associated with increased morbidity and mortality, but current surveillance strategies are inadequate for early detection. Prior studies indicate that tissue glycosylation is altered in thyroid cancer, but the utility of serum glycosylation in thyroid cancer surveillance remains unexplored. We therefore assessed the potential utility of altered serum glycomic profile as a tumor‐specific target for disease surveillance in recurrent thyroid cancer.

**Experimental design:**

We employed banked serum samples from patients with recurrent thyroid cancer post thyroidectomy and healthy controls. N‐glycans were enzymatically released from serum glycoproteins, labeled via permethylation, and analyzed by MALDI‐TOF mass spectrometry. Global level and specific subtypes of glycan structures were compared between patients and controls.

**Results:**

We evaluated 28 independent samples from 13 patients with cancer recurrence and 15 healthy controls. Global features of glycosylation, including N‐glycan class and terminal glycan modifications were similar between groups, but three of 35 individual glycans showed significant differences. The three glycans were biosynthetically related biantennary core fucosylated N‐glycans that only varied by the degree of galactosylation (G0F, G1F, and G2F; G: galactose, F: fucose). The ratio of G0F:G1F that captures reduced galactosylation was observed in patients samples but not in healthy controls (*p* = 0.004) and predicted thyroid cancer recurrence (AUC = 0.82, CI 95% = 0.64–0.99).

**Conclusions:**

Altered N‐glycomic profile was associated with thyroid cancer recurrence. This serum‐based biomarker would be useful as an effective surveillance tool to improve the care and prognosis of thyroid cancer after prospective validation.

## Abstract 2

Cellular transformation is associated with alterations in cell surface carbohydrates or glycans. However, the role of glycosylation in thyroid cancer recurrence remains unexplored. Using N‐glycomics of total patient serum we identified a glycan‐based biomarker that is associated with thyroid cancer recurrence.

## 1 INTRODUCTION

Thyroid cancer is a major public health concern with approximately 50,000 new cases in the United States in 2020. It is the fifth most common cancer in women, the most commonly diagnosed cancer in young adults of ages 15–29, and one of the cancers with the fastest rise in rates of diagnoses with a concomitant increase in cancer mortality.^1^, ^2^ The differentiated thyroid cancer (DTC) subtypes account for the majority of the rising cases.^2^

The mainstay of treatment for thyroid cancer remains surgical resection with thyroidectomy especially for the vast majority of DTC. Although many patients do well with a 5‐year overall survival (OS) of 98% following surgery, a significant proportion of patients recur with local and or distant spread of the disease. The 5‐year OS with recurrent disease drops to 78% and 63%, respectively, for local and distant recurrence and this accounts for the rising mortality rates.^1^, ^2^ Identifying patients with high‐risk feature who will recur after surgery is crucial as it determines the need and type of adjuvant therapy, such as radioactive iodine (RAI), external beam radiation, thyroid stimulating hormone (TSH) suppression, as well as the surveillance strategy. Currently, the American Thyroid Association clinicopathologic risk stratification system is used to classify DTC into low, medium, and high risk for persistence/recurrence with all but low risk patients recommended for RAI.^3^ However, this ATA risk stratification remains inadequate in terms of overall accuracy for quantifying the risk of recurrent disease: approximately 10% of low risk patients, 50% of intermediate risk patients, and 70% of high risk patients develop recurrent disease,^3^, ^4^, ^5^, ^6^, ^7^ and other biomarkers and metrics (e.g. thyroglobulin, TNM staging, lymph node involvement, mutational status, and/or the degree of vascular invasion) have failed to improve prediction and detection of disease recurrence in individual patients.^3^

Altered glycosylation has emerged as a hallmark of cancer and unique glycosylated proteins currently serve as clinical tumor markers, such as CA19‐9, CA‐125, CA15‐3, CEA, and AFP.^8^, ^9^ These include N‐ and O‐linked glycans, which are present on over 80% of cell surface proteins and regulate cell‐signaling, immunity, protein turnover/stability, and cellular/molecular recognition.^8^, ^9^, ^10^, ^11^, ^12^ N‐glycans, in particular, are built off of a pentasaccharide core, Man_3_GlcNAc_2_. Depending on the extent of processing and modification, N‐glycans form high‐mannose/oligomannose structures terminating exclusively in mannose, complex structures terminating in non‐mannose monosaccharides, or a combination of both to form hybrid structures. Altered serum glycosylation profile results from direct release of glycans from diseased tissues into serum or from indirect rewiring of glycans attached to acute phase reactants.^8^, ^13^, ^14^

Prior studies analyzing glycosylation in thyroid cancer have primarily assessed specific glycoproteins or glycans in low‐risk patients.^15^, ^16^, ^17^, ^18^, ^19^, ^20^, ^21^, ^22^, ^23^, ^24^, ^25^, ^26^, ^27^, ^28^, ^29^, ^30^, ^31^ Few studies have analyzed total thyroid^32^ or serum glycans,^33^ and none have specifically analyzed high‐risk patients with recurrent disease. Nonetheless, prior studies have found altered N‐glycosylation in thyroid neoplasia, suggesting that these or related changes may also be useful in identifying thyroid cancer recurrence.

We performed matrix‐assisted laser desorption/ionization (MALDI)‐time of flight (TOF) mass spectrometry‐based total serum N‐glycomics of healthy controls versus recurrent thyroid cancer patients, characterized individual and global patterns of serum glycoprotein N‐glycosylation, and discovered an association between thyroid cancer recurrence and serum N‐glycosylation. Our study represents the first analysis of glycosylation in recurrent thyroid cancer and identifies a promising non‐invasive biomarker to predict high risk patients that may benefit from adjuvant therapy or aggressive surveillance following thyroidectomy.

## 2 MATERIALS AND METHODS

### 2.1 Patient samples

We employed banked serum samples previously collected from patients with differentiated thyroid cancer or non‐cancer controls without thyroid disease and stored at −80°C until thawed for glycan analysis. Samples were originally collected prospectively following patient assent under an institutional review board‐approved protocol as previously described.^34^ Patient characteristics were previously described.^34^

### 2.2 N‐glycan purification

N‐glycans were analyzed as previously described with some modifications.^35^ N‐glycans were released from serum. 20 μl human serum was mixed with 20 μl 10X denature buffer (New England Biolabs) and water was added to bring the final reaction volume of 200 μl, which was boiled for 10 min and then cooled to room temperature. PNGaseF digestion mixture (25 μl NP‐40, 25 μl digestion buffer, and 1 μl PNGase F) was added and incubated at 37C for 16 h, an additional 1 μl PNGaseF was added, and then incubated to a total time of 24 h. The reaction mix was centrifuged at 16,000 *g* for 5 min, the supernatant collected and added to an activated Sep‐Pak C18 column, followed by an activated carbograph column, lyophilized, and dried overnight.

### 2.3 N‐glycan permethylation

After purification, 0.5 ml NaOH in DMSO 50% slurry (1 pellet NaOH in 1 ml DMSO) was added to the sample, followed by 0.2 ml CH_3_I, which was then shaken for 1 h. 500 μl water and then 500 μl chloroform were added, then centrifuged at 700 *g* for 2 min. The upper layer was descanted and discarded. The lower phase was washed with 500 μl 0.5 M sodium chloride 3 times, while decanting and discarding the upper phase each time. The lower phase was then dried by speed vac.

### 2.4 Mass spectrometry

After permethylation, the sample was reconstituted in 20 μl 50% methanol. 1 μl sample was directly analyzed by MALDI‐TOF‐MS, while 10 μl sample were further purified with C18 Sep‐Pak column and eluted with 15%, 30%, 50%, 75% acetonitrile, dried by speed vac, reconstituted in 20 μl 50% methanol, with 1 μl sample analyzed by MALDI. Ultimately, the directly analyzed or acetonitrile fractionated specimen containing N‐glycans was reported depending on which had greater signal‐to‐noise. 0.5 μl of sample was added to 0.5 μl of DHB matrix (20 mg/mL 2,5‐dihydroxybenzoic acid in 80:20 (v/v) methanol: water) on Bruker AnchorChip target plate, dried, and analyzed in positive‐ion reflector mode using Ultraflex‐II TOF‐TOF system (Bruker Daltonics) and flexControl software.

### 2.5 Glycan analysis

MS peaks were automatically extracted with flexControl software from all samples and compared to a list of known N‐glycan compositions corresponding to molecular ions m/z of [M + Na]^+^. Unmatched peaks were discarded, while matched peaks were manually re‐analyzed by MALDIquant package^36^ in R‐project to confirm appropriate isotopic distribution for a representative subset (at least 1 spectra for each glycan). Those peaks that passed this two‐point quality control were considered for final analysis. For global analysis in Figure 2, each peak within a sample was normalized to the max signal intensity from a given spectra, whether or not it was a glycan, and the intensity of peaks corresponding to a given feature (e,g. oligomannose) were summed and reported as a percentage of all glycan peaks summed. For analysis of individual glycans in Figure 3, each glycan within a sample was normalized to the max signal intensity from a glycan within that spectrum, thus setting the most abundant glycan in each sample to 100%. Only those glycans present in at least 3 replicate samples each for controls and patients were analyzed. For comparing the ratio of intensities of G0F:G1F for Figure 4, this ratio was calculated for each sample and then compared across samples. Glycan cartoons were created with GlycoGlyph.^37^

### 2.6 Statistics

Analysis was performed with ANOVA with SIDAK's test for multiple comparisons, unpaired two‐tailed *t*‐tests, or Mann–Whitney test, depending on whether assumptions for a parametric test were met. A receiver operating characteristic (ROC) curve was generated by standard methods with the ratio of intensities of G0F:G1F. Select sensitivities and specificities for given cut‐offs were extracted following manual ROC curve inspection. Graphpad Prism 7.0 was used for data analysis. *p* ≤ 0.05 was considered statistically significant and denoted with an asterisk or otherwise as not significant (ns).

## 3 RESULTS

### 3.1 Global glycan analysis

Serum glycomics offers a unique opportunity to identify non‐invasive biomarkers of disease. We analyzed serum N‐glycans from patients with differentiated thyroid cancer who recurred following thyroidectomy (*n* = 13) and control individuals without cancer or thyroid disease (*n* = 15). N‐glycans were enzymatically released from serum glycoproteins, labeled via permethylation, and analyzed by matrix‐assisted laser desorption/ionization‐time of flight (MALDI‐TOF) mass spectrometry. Glycan structures were inferred from MS data and biosynthetic pathways.

**FIGURE 1:**
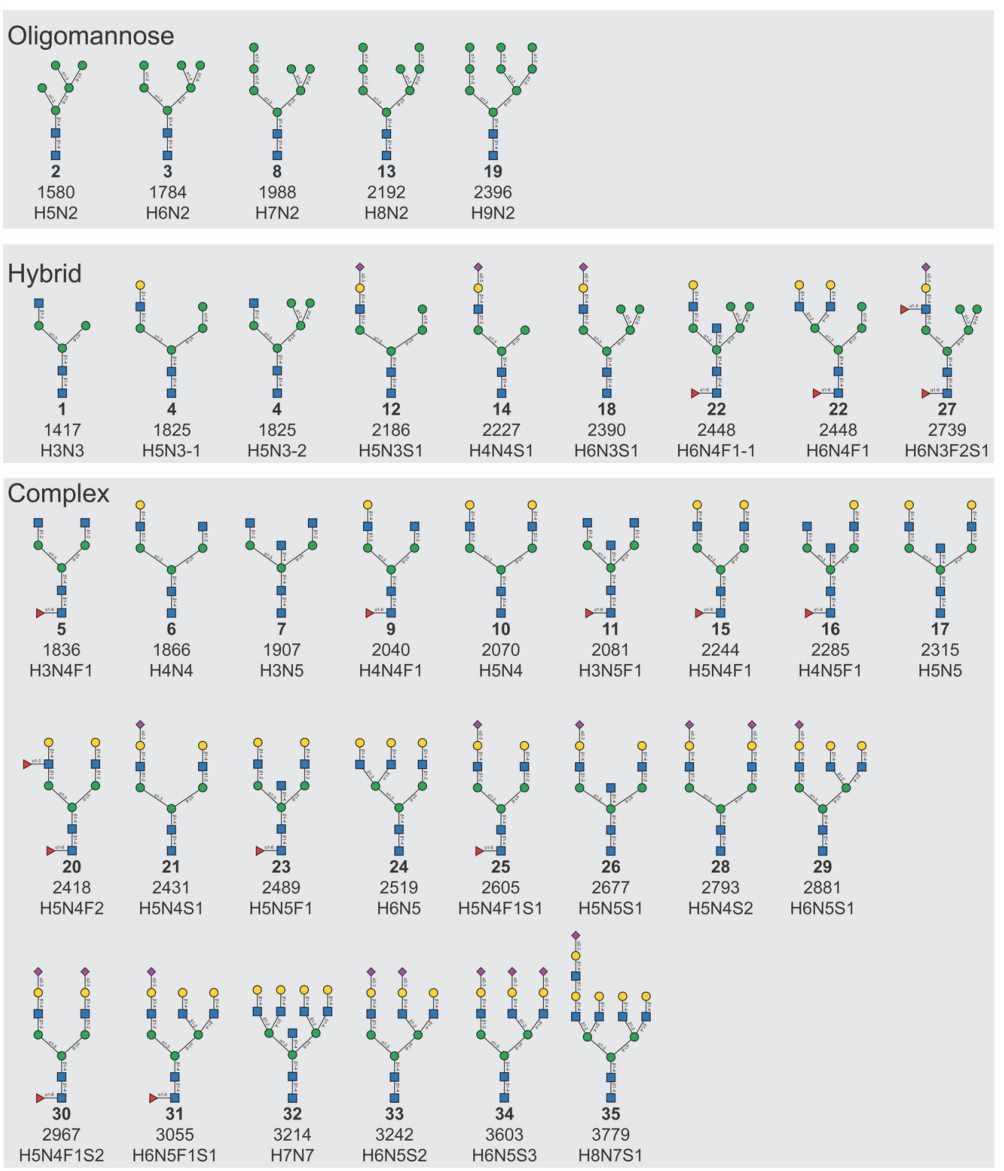
Structures of N‐glycans in our cohort. All 35 serum N‐glycans identified from patients (*n* = 13) and controls (*n* = 15) are listed. Structures are inferred from mass spectrometry compositions and biosynthetic knowledge. H: Hexose, N: HexNAc, S: Neu5Ac, F: fucose.

**FIGURE 2:**
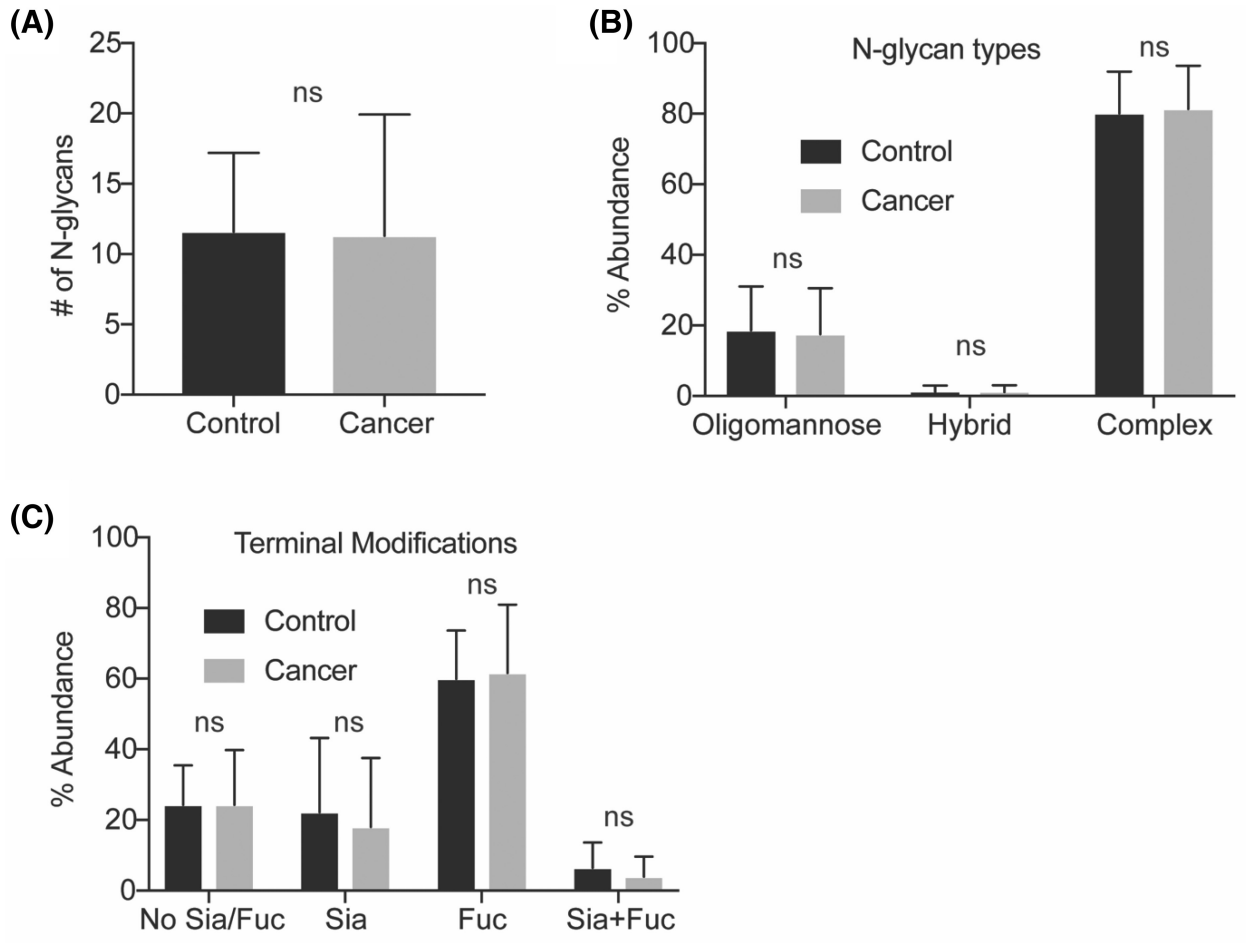
Global analysis of N‐glycans from controls versus patients. (A) The number of N‐glycan compositions, (B) N‐glycan type, and (C) N‐glycan monosaccharide modifications were compared in serum from thyroid cancer patients (*n* = 13) versus controls (*n* = 15). *p ≤ 0.05 from unpaired two‐tailed t test (A) and ANOVA with Sidak's test for multiple comparisons (B, C); ns, not significant.

In total, we identified 35 unique glycan compositions, including five oligomannose, nine hybrid, and twenty‐three complex‐type N‐glycans (Figure 1 and Figure S1). The total number of N‐glycans (mean 11 per sample) did not differ between groups (Figure 2a). To assess potential changes in glycan processing, we compared the abundance of N‐glycan types (oligomannose, hybrid, complex). No difference was observed between patients and controls (Figure 2b). Overall, complex‐type was the most abundant (81%), followed by oligomannose (18%) and hybrid‐type N‐glycans (1%) (Figure 2b).

We next assessed terminal N‐glycan modifications, including sialylation, fucosylation, dual modification, or no modification (Figure 2c). Again, no difference was observed between patients and controls. Fucose (61%) was the most common modification, followed by no sialic acid or fucose modification (24%), sialic acid modification (20%), and dual modification with fucose and sialic acid (5%) (Figure 2c). Collectively, no difference in global glycosylation patterns was seen in cancers versus controls.

### 3.2 Analysis of individual glycans

**FIGURE 3:**
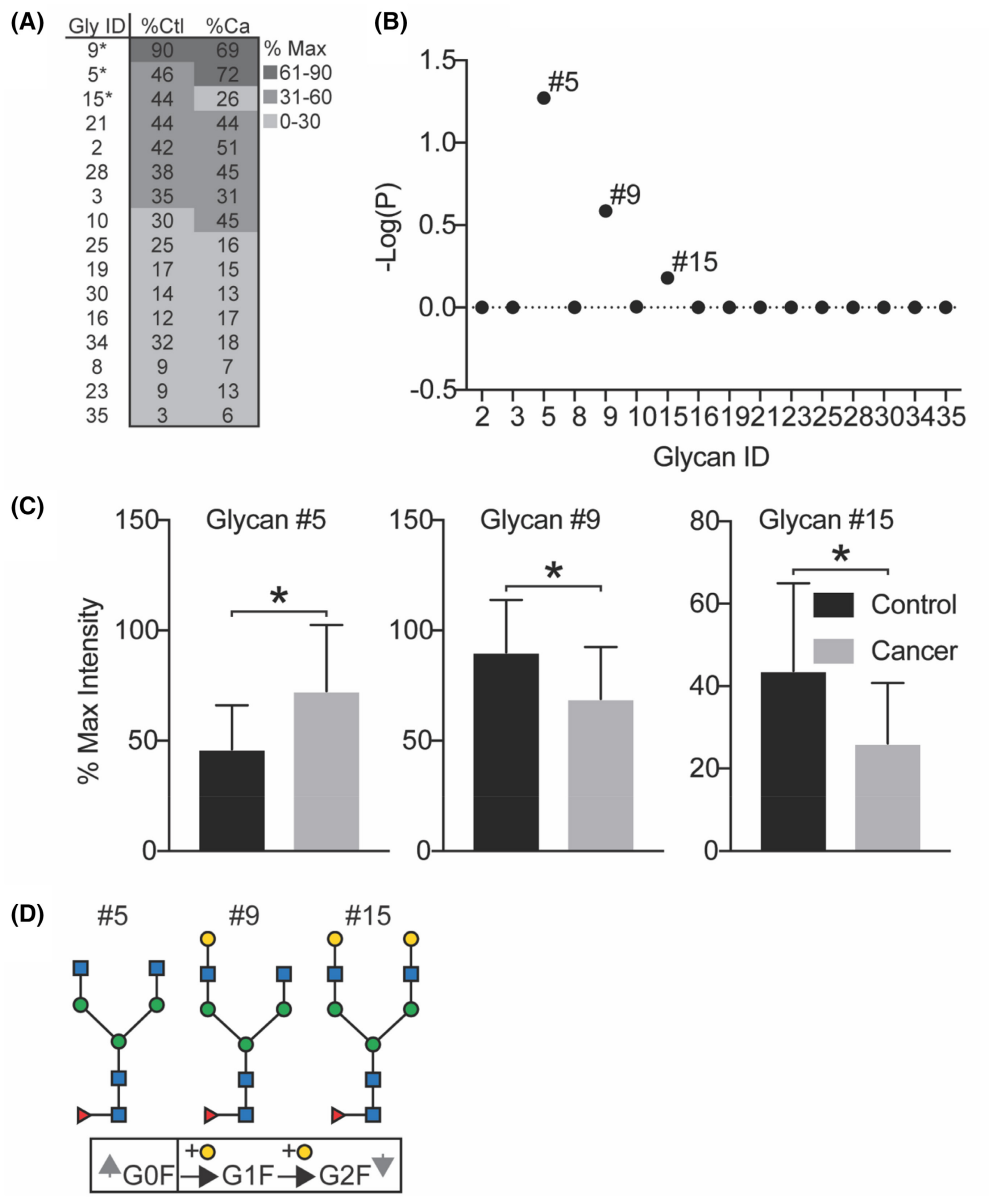
Analysis of individual N‐glycans from controls versus patients. (A) Heatmap of mean MS intensities corresponding to glycan IDs in descending order of control glycans; only glycans also analyzed in B are shown; *significant *p*‐values obtained from analysis for C. (B) Negative log of *p*‐values was plotted for all glycans present in at least 3 samples each from control (*n* = 15) and cancer patients (*n* = 13). (C) Glycans that differed from the median were further analyzed and (C) depicted. (D) Biosynthetic pathway of significantly altered glycans is depicted; up and down arrows correspond to changes observed in cancer (more G0F, less G1F and G2F in cancer). G0F: biantennary core fucosylated N‐glycan; G1F: monogalactosylated biantennary core fucosylated N‐glycan; G2F: bigalactosylated biantennary core fucosylated N‐glycan. *p ≤ 0.05 from ANOVA with Sidak's test for multiple comparisons (B) and unpaired two‐tailed *t* test (A, C).

Analysis of global N‐glycan features can fail to capture finer biosynthetic details. To address this, we analyzed changes in individual N‐glycans. We evaluated 16 of 35 compositions that were present in at least 3 controls and 3 patients (Figure 3a). Eight of these 16 glycans had ≥30% abundance in either control or cancer samples with all but Glycan #15 (G2F) abundant in both groups (Figure 3a). These abundant eight glycans included biantennary (G0F, G1F, G2F, G2, G2S1, G2S2) and oligomannose (Man5, Man6) N‐glycans.

To further compare potential changes in cancer recurrence, we constructed a Manhattan Plot (negative log of *p*‐values) for all 16 glycans (Figure 3b). Three glycans, #5, #9, and #15, clearly separated from baseline (Figure 3b) and statistically differed in controls versus patients (Figure 3c, *p* < 0.05 for all comparisons, two‐tailed student's *t*‐test). Notably, these glycans are biosynthetically related, with varying degrees of galactosylation on a biantennary N‐glycan with a core fucose residue (Figure 3d). Glycan #5 is agalactosylated (G0F), glycan #9 is monogalactosylated (G1F), and glycan #15 is digalactosylated (G2F). Compared to controls, cancer patients have reduced galactose with a corresponding increase in glycan #5 (G0F) and a corresponding decrease in glycan #9 (G1F) and glycan #15 (G2F) (Figure 3c,d). Hence, in contrast to global glycosylation features, cancer recurrence is associated with a reduction of galactose on biantennary N‐glycans.

### 3.3 Galactosylation as a biomarker for thyroid cancer

**FIGURE 4:**
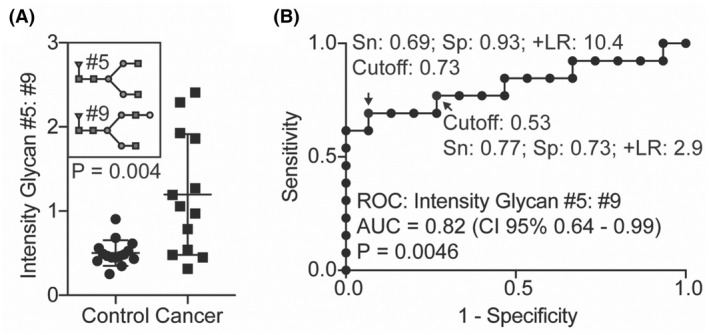
Biantennary N‐glycan galactose as a biomarker for recurrent thyroid cancer. (A) The ratio of mass spectrometry intensities of G0F:G1F for control (*n* = 15) versus cancer patients (*n* = 13); (B) the ROC curve for G0F:G1F with sensitivities and specificities for manually selected cutoffs are shown; and P values from Mann–Whitney (A) and ROC curve (B) are shown.

To develop a biomarker that fully captures the shift in galactosylation, we compared the ratio of glycan #5 to glycan #9 (G0F:G1F, agalactosylated:monogalactosylated). Since these glycans are extremely abundant, G0F:G1F should be readily measurable for most patients. Glycans#5 and #9 were present in all 28 of our samples. In contrast, glycan #15, which like glycan #9 was also reduced in cancer, was absent from 3 of 28 samples. Using this ratio of G0F:G1F, patients were clearly distinguished from controls with an increase of G0F:G1F in cancer reflecting reduced galactosylation (Figure 4a, *p* = 0.004, Mann–Whitney).

We generated a receiver operating characteristic (ROC) curve of G0F:G1F to evaluate test characteristics and obtained an area under the curve (AUC) of 0.82 (95% confidence interval, CI, of 0.64–0.99, *p* = 0.0046) (Figure 4b). Two cutoffs were manually selected either to maximize specificity (G0F:G1F = 0.73) or to optimize both sensitivity and specificity (G0F:G1F = 0.53). A cutoff of 0.73 yielded a sensitivity of 0.69, specificity of 0.93, a positive likelihood ratio of 10.4 (+LR, 95% CI of 1.51–71), and a negative likelihood ratio of 0.33 (−LR, 95% CI of 0.14–0.75), while a cutoff of 0.53 yielded a sensitivity of 0.77, specificity of 0.73, +LR of 2.88 (95% CI of 1.18–7.03), and a ‐LR of 0.31(95% CI of 0.11–0.89) (Figure 4b). Thus, we defined G0F:G1F as a potential biomarker to predict thyroid cancer recurrence.

## 4 DISCUSSION

We performed serum N‐glycomics on patients with differentiated thyroid cancer who recurred following thyroidectomy and identified changes in glycosylation that predict cancer recurrence. Compared to controls, patients had reduced galactosylation of a specific core fucosylated, biantennary N‐glycan. Importantly, the ratio of agalactosylated (G0F) to monogalactosylated (G1F) species was able to predict recurrence with an AUC of 82% and sensitivities and specificities over 70%.

Although the majority of patients with thyroid cancer have good outcomes, a small subset of patients has recurrent disease and poor survival. Efforts to identify these patients have focused on clinical, histologic, and more recently genomic‐based biomarkers. However, these markers are suboptimal in performance and often fail to adequately identify high‐risk patients who could benefit from adjuvant intervention. On the other hand, failure to accurately identify low‐risk patients results in such patients receiving unnecessary adjuvant therapies, with associated adverse effects and avoidable costs.

Thyroglobulin is the most widely used tumor marker for differentiated thyroid cancer, but has several limitations. Thyroglobulin is not a reliable marker in the approximately 25% of DTC patients with anti‐thyroglobulin antibodies^38^, ^39^; exhibits significant inter‐test heterogeneity, making cross‐lab comparisons challenging; is less reliable under TSH suppression; and performs better after combined thyroidectomy followed by RAI ablation rather than thyroidectomy alone. A meta‐analysis of 9094 patients found a sensitivity and specificity of thyroglobulin to detect persistent thyroid cancer of 96% and 95% after thyroid hormone withdrawal, 93% and 88% after TSH stimulation, 78% and 98% on thyroid hormone, and 76% and 97% in patients who did not undergo remnant ablation.^40^ As an example, among 495 low‐risk TNM stage I and II patients with a median of 11.6 years of follow‐up, thyroglobulin was elevated 173 times and, alone, was only able to detect recurrence in 23 of 44 patients.^41^ Since thyroglobulin is a maker of thyroid tissue, its presence after surgery and thyroid ablation suggests the presence of thyroid cancer, which may not yet be detectable on imaging. This conceptually contrasts from a biomarker that can predict persistence/recurrence independent of the existence of current, detectable disease.

We observed a sensitivity, specificity, +LR, and −LR of 69%, 93%, 10.4, and 0.33 with a G0F:G1F cutoff of 0.73 versus 77%, 73%, 2.88, 0.31 with a G0F:G1F cutoff of 0.53. This compares to a sensitivity, specificity, +LR, −LR of 78%, 98%, 34, 0.23 of thyroglobulin by meta‐analysis in patients post‐thyroidectomy who underwent remnant ablation (LR calculated from manuscript based on reported prevalence of 0.23, *n* = 1613 with immunometric assay).^40^ The test characteristics of G0F:G1F for our 28 patients is numerically slightly reduced compared to 1613 patients evaluated in the metanalysis (sensitivity 69 or 77% versus 78%, specificity 93 or 73% versus 98%, +LR 10.4 or 2.9 versus 34, −LR 0.33 or 0.31 versus 0.23).^40^ However, G0F:G1F is distinct from thyroglobulin, so may address limitations of thyroglobulin, such as patients with thyroglobulin antibody, patients receiving thyroid hormone, or to predict risk of cancer recurrence prior to recurrence. Further, G0F:G1F could be combined with thyroglobulin, US, and/or ATA risk stratification to predict risk of recurrence or to identify recurrent/persistent disease.

Prior glycosylation studies analyzed unselected, mostly low risk differentiated thyroid cancers versus controls and assessed specific glycoproteins isolated from thyroid tissue,^15^, ^16^, ^17^, ^18^, ^19^, ^20^, ^21^, ^22^, ^23^, ^24^ binding of lectins and antibodies,^25^, ^26^, ^27^, ^28^ expression of glycan binding proteins,^15^ and expression of glycosyltransferases.^29^, ^30^, ^31^ Only two studies performed glycomics, analyzing all glycans present in a sample in an unbiased manner. One such study analyzed samples of normal and malignant thyroid tissues from 23 patients (control tissue was adjacent normal from PTC tissue block) and found an increase in complex, biantennary, fucosylated, galactosylated, and sialylated glycans in the tumor samples.^32^ Another study analyzed serum samples and reported reduced bisecting GlcNAc and increased galactosylation and sialylation on IgG1 (*n* = 138 thyroid cancer, *n* = 735 healthy controls).^33^ Collectively, compared to controls, these older studies found increased galactose in both thyroid cancer tissue and serum. In contrast to these studies of mostly low‐risk thyroid cancer, we found that reduced galactose in serum is associated with cancer recurrence, suggesting that mechanisms for thyroid glycosylation in thyroid transformation differ from those that drive thyroid cancer recurrence. Although reduced serum galactose has been observed in autoimmune and inflammatory disease, patients with autoimmune thyroiditis have a reduced risk of thyroid cancer recurrence, suggesting that the reduction in galactose in high risk patients in our study does not clearly reflect a change in the immune milieu or selection for a pro‐inflammatory state.^42^, ^43^, ^44^, ^45^, ^46^, ^47^, ^48^, ^49^

Our study has several potential limitations. We analyzed a relatively small number of patients with 13–15 samples per group. Nonetheless, these samples were collected prospectively,^34^ and we were able to identify clear differences between controls and patients. Although we stored these samples at −80°C for 5~10 years, patients and controls were stored over similar time periods, the major peaks were biosynthetically relevant structures rather than degradation products, the structures in controls and patients compared well with prior studies, and serum glycans have been shown to be stable over long periods of time, even at room temperature if properly stored.^50^, ^51^ Additionally, although we only analyzed samples at single time points, the serum glycome in an individual is stable over time.^52^ Another potential limitation is that we only compared patients with recurrent disease to non‐cancer controls rather than to patients who did not recur. Nonetheless, prior studies have found that serum and thyroid tissue from unselected thyroid cancer patients, most of whom would not have recurrent disease, had increased rather than reduced galactose,^32^, ^33^ indicating that the change we observed in cancer recurrence is likely to be unique to recurrence and does not simply reflect a general change in thyroid cancer. Although we evaluated the serum glycome in patients who recurred, the serum glycome reflects tumor‐dependent and independent regulation and may predict recurrence prior to structural disease. Another question is whether the change in serum glycosylation we observed reflects a change due to surgery rather than a change due to cancer recurrence. Although the effect of surgery on the serum glycome is not well studied, limited data from non‐thyroid surgery suggest that serum glycome can change for up to several weeks post‐surgery but normalizes by one year.^53^, ^54^ The median time from surgery to recurrence was 1.6 years in our original cohort,^34^ suggesting that any post‐surgical changes in glycosylation would have normalized and thus not the major contributor to our findings.

Prior strategies to identify high‐risk thyroid cancers are limited. Our discovery of a non‐invasive glycomic biomarker for disease recurrence could significantly impact patients with differentiated thyroid cancer. Additionally, the identification of a glycan‐based biomarker that identifies patients at high risk of thyroid cancer recurrence suggests that glycosylation is not simply a non‐specific marker of cellular transformation, but rather reflects the complex biology associated with distinct disease phenotypes and thus could be leveraged for personalized medicine.

## AUTHOR CONTRIBUTIONS

MRK and TKO designed the project. MRK, YL, and XS performed the experiments. MSH helped with original sample collection. MRK analyzed the data. MRK, TKO, YL, DFS, and XS oversaw experiments. MRK wrote the manuscript. TKO edited the manuscript. All authors reviewed and approved the manuscript.

## FUNDING INFORMATION

This study was supported in part by the Emory Comprehensive Glycomics Core (ECGC), which is subsidized by the Emory University School of Medicine and is one of the Emory Integrated Core Facilities. Sample collection was supported by a pilot grant mechanism as part of the NIH SPORE grant under Award Number P50CA128613 and by the Tissue Procurement and Pathology Shared Resource of Winship Cancer Institute of Emory University, Atlanta, GA 303022 under the NCI Cancer Center Support Grant Award Number P30CA138292. Additional support was provided by the National Center for Advancing Translational Sciences of the National Institutes of Health under Award Number UL1TR000454 and the cancer center support grant to the UPMC Hillman Cancer Center at University of Pittsburgh under award number 5P30CA047904. The content is solely the responsibility of the authors and does not necessarily reflect the official views of the National Institutes of Health.

## CONFLICT OF INTEREST

MRK, TKO, YL, DFS, XS, and MHS declare no competing financial interest.

## INSTITUTIONAL REVIEW BOARD STATEMENT

Sample collection on study was approved by the Emory University Institutional Review Board. The study was conducted in accordance with the Declaration of Helsinki and approved by the Institutional Review Board of Emory University (WCI1792‐10).

## INFORMED CONSENT STATEMENT

Patient consent was waived by the IRB due to “minimal risk” nature of the study. An informational document summarizing the purpose of the study was provided to study participants prior to blood sample collection.

## PRECIS

Neoplastic transformation is associated with alterations in cell surface carbohydrates or glycans; however, the role of glycosylation in cancer recurrence and thyroid cancer recurrence in particular is unexplored. We performed N‐glycomics of total patient serum and identified a glycan‐based biomarker that is associated with recurrent thyroid cancer.

## Supporting information


Figure S1

## Data Availability

N/A
