# Supplementary figures and images for: Serum glycomic profile as a predictive biomarker of recurrence in patients with differentiated thyroid cancer
Source: Cancer Med. 2022 Nov 27;12(6):6768–77. doi: 10.1002/cam4.5465 (PMC10067050; doi:10.1002/cam4.5465)

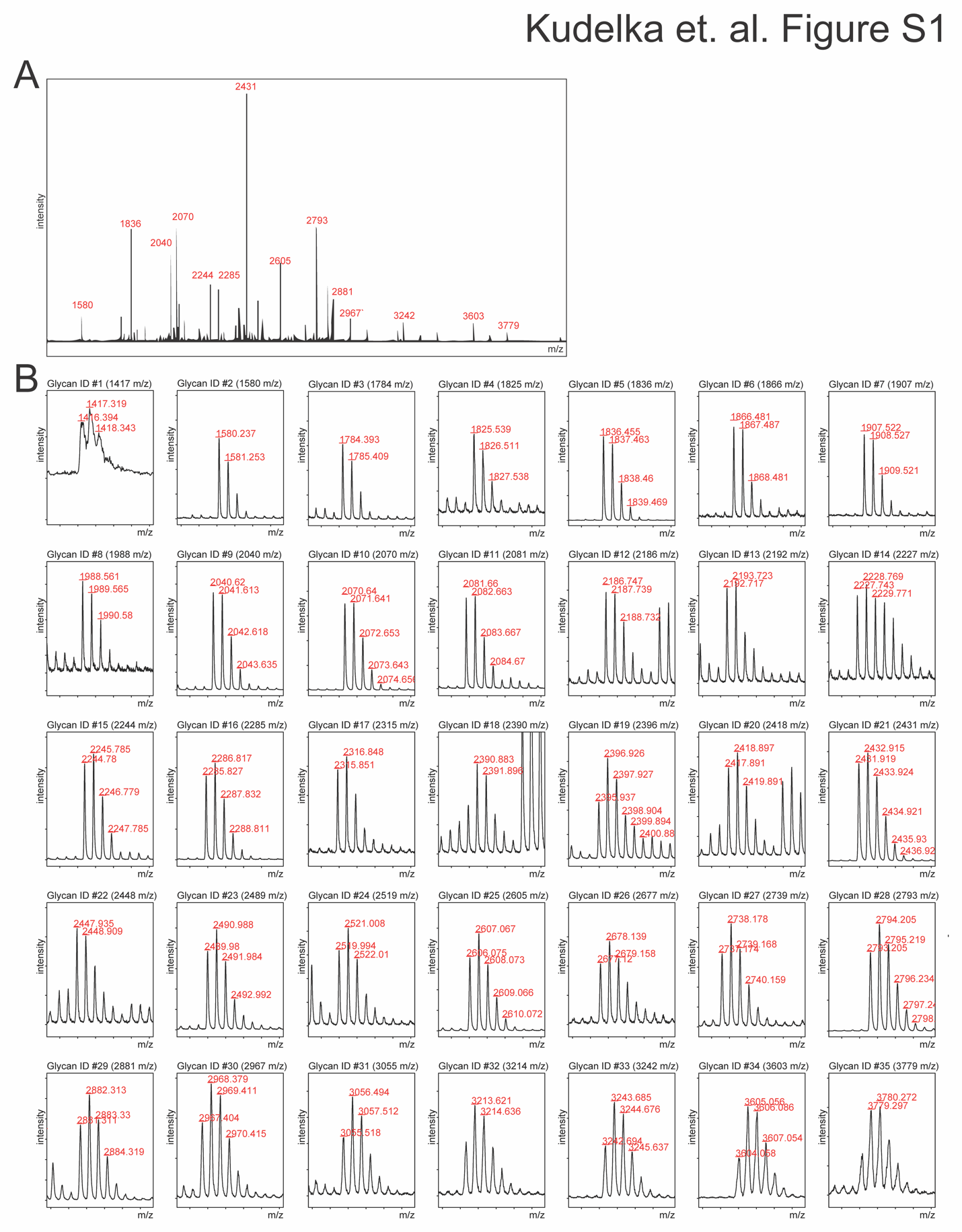


**Figure S1. Mass spectra of N-glycans**

Supplement: Supplementary file 1 — Figure S1 [file CAM4-12-6768-s001.docx]
